# Next Generation Cancer Protection: The Bivalent HPV Vaccine for Females

**DOI:** 10.5402/2011/457204

**Published:** 2011-11-02

**Authors:** Diane M. Harper, Stephen L. Vierthaler

**Affiliations:** Center of Excellence, Women's Health, University of Missouri-Kansas City School of Medicine, 7900 Lee's Summit Road, Kansas City, MO 64139, USA

## Abstract

Nearly a half a million women throughout the world develop cervical cancer every year Parkin and Bray (“Chapter 2. The burden of HPVrelated cancers,” Vaccine, vol. 24, no. 3, pp. S11–S25, 2006); 80% of these women are in countries without a quality-assured cytology screening program. It is in this setting that Cervarix could reduce the incidence of cervical cancer to about 9.5/100,000 women. New evidence indicates that this might be able to be accomplished with a single dose of Cervarix, a great advantage to public health implementation programs Kreimer et al. (“Proof-of-principle evaluation of the efficacy of fewer than three doses of a bivalent HPV16/18 vaccine, *The Journal of the National Cancer Institute*, vol. 103, no. 19, pp. 1444–1451, 2011). In countries with screening programs, adenocarcinoma is the most difficult to detect and treat with later-stage presentation and higher mortality Smith et al. (“The rising incidence of adenocarcinoma relative to squamous cell carcinoma of the uterine cervix in the United States—a 24-year population-based study,” *Gynecologic Oncology*, vol. 78, no. 2, pp. 97–105, 2000) and Gunnell et al. (“A longitudinal Swedish study on screening for squamous cell carcinoma and adenocarcinoma: evidence of effectiveness and overtreatment,” *Cancer Epidemiology Biomarkers and Prevention*, vol. 16, no. 12, pp. 2641–2648, 2007). With additional cross-protection to HPV 31, 33, and 45 and protection against HPV 16 and 18 lasting at least 9.4 years, Cervarix may reduce adenocarcinomas in screened populations by more than 90%. This paper will detail the evidence about the efficacy, immunogenicity, and safety of Cervarix in the studied populations contrasting public health goals with individual health options.

## 1. Introduction

Cervical cancer is unequivocally initiated by a human papillomavirus (HPV) infection, but much less than 1% of HPV infections progress to cervical cancer [1]. This inconsistency has made the cervix the site most studied of all HPV infections. The first associations of HPV infections with cervical cancer specimens were made by zur Hausen [2] and Meisels and Fortin [3]. Multiple case control, cohort, and, finally, prospective longitudinal studies have provided much detail in concert with biochemical mechanistic studies about the steps of HPV infection and where they branch to oncogenesis. This paper will detail the efficacy, immunogenicity, and safety of Cervarix as it pertains to interruption of the infection process to prevent cervical cancer.

### 1.1. Low- versus High-Risk HPV Types

The carcinogenic risk potential of the mucosal alpha genus HPV types is categorized as low or high-risk. Low- and high-risk types were so classified based on their propensity to be associated with cervical cancer. High risk types had odds ratios of association with cervical cancer in the hundreds, much higher than the odds ratios found linking tobacco use and lung cancer [4]; low-risk types had odds ratios around one, indicating very little to no risk of malignant transformation [5]. The designation of high-risk types has been inconsistent in the International Agency for Research on Cancer (IARC) monographs, but has most recently included four species (5, 6, 7, and 9) of the alpha genus of papillomaviruses. The twelve HPV types considered carcinogenic by current evidence include one from species 5 (HPV 51), one from species 6 (HPV 56), four from species 7 (HPV 18, 39, 45, and 59), and six from species 9 (HPV 16, 31, 33, 35, 52, and 58) [6]. Of these types, HPV 16 and HPV 18 are the most aggressively oncogenic [7].

### 1.2. Clinical Manifestations of Viral Infection

HPV presents in a variety of clinical appearances classified in two categories: benign and oncogenic. One of the most common differentiating factors is the expression of the E2 protein. If E2, an early expressed HPV protein, is not disrupted, then HPV infection most often remains episomal within the epithelial basal cell. As an episomal infection, benign forms such as condyloma and cervical intraepithelial neoplasia grade 1 (CIN 1) are clinically seen, despite the type of HPV that is causing the infection [8]. If the HPV genome is disrupted, which happens most often after integration within the human chromosome, then oncogenesis can proceed and CIN 2/3 can occur. Only those persistent high-risk HPV infections have the opportunity to progress to CIN 3, yet less than half of persistent infections do progress [9]. There is very slow progression from CIN 3 to invasive cancer [10] with some CIN 3 regressing to normal epithelium [11].

The validity of CIN 2 being a cancer precursor is uncertain due to high misclassification rates [12], and poor intra- and interobserver reproducibility in diagnosis [13], as well as high regression rates to normal in young women [14]. While there is considerable scientific debate around the CIN 2 classification, clinically CIN 2 is considered a cancer precursor and standardly treated as such. Therefore, the HPV vaccine trials include CIN 2 as a cancer precursor endpoint.

### 1.3. Time Sequence of Events

The natural history of HPV infections in the cervical epithelium in vivo cannot be detected unless the infection is actively replicating. Most actively replicating HPV infections in postpubertal females must reach a significant viral threshold to produce a cytologically detectable lesion [15, 16]. Hence, there is a time delay from infection to detection of cervical cytologic abnormalities. Most are detected on average between 10 and 44 months after a HPV 16/18 infection and between 19 and 45 months for all other high-risk HPV infections, while low-risk HPV infections can be detected by cytology on average 10 to 55 months after infection in young women [17–19]. While HPV infection is very common, with nearly a fifth of all women of screening age infected, less than 8% of screened women have associated cytologic abnormalities [20–23]. Cell-mediated immunologic clearance controls most infections, but not all. 70% of cytologically expressed infections will clear within one year, and more than 90% within three years [9, 24]. Of those cytologically expressed infections that do not clear, only half progress to precancerous cervical intraepithelial neoplasia grade 2 or 3 (CIN 2/3) [9]. It takes five years for 20% of the large visible CIN 3 lesions to progress to invasive cancer, and after 30 years, only 40% of CIN 3 lesions have become cervical cancer [10]. This is, on average, a very slowly progressing cancer for which screening has been very successful in preventing [25].

### 1.4. Natural Infection Does Not Protect against Future HPV Infections

The natural immunologic response of HPV infections is poorly understood, and its meaning is vigorously debated. Most of the data are from studies of young sexually active women. About half of the young women with oncogenic HPV infections seroconvert. Of those who do, seroconversion takes months to detect, usually at low antibody titers [26–29] which are most often insufficient to prevent reinfection with the same HPV type [30–32]. There is one study in young women which shows that women with >60 EU/mL of anti-HPV 16 have about half of the incident HPV 16 infections that women with lower or nondetectable titers develop [31]. Whether this represents a threshold value for immunoprotection or whether this is an artifact of antibody measurement is unknown.

HPV does not cause a viremia, and hence, as a natural infection, it has very little chance of initiating an anamnestic response of prior primed memory B cells. Immunologically, HPV does not cause cell lysis in either the episomal or integrated infection format. HPV does not incite an inflammatory response at the time of infection or reinfection; there are no proinflammatory cytokines released, and there is poor exposure and activation of the epithelial antigen-presenting cells and Langerhans cells [33]. This lack of immune recognition allows new infections of the same or different HPV types to be established.

### 1.5. HPV Infection by Age

HPV infection is documented by viral DNA detection in the cervicovaginal epithelium or by serologic evidence. Both DNA detection and serologic assays have detected high-risk HPV infections in all ages of females with the incidence as high as 10% in those under 11 years of age [34–39]. Peak prevalence of high-risk HPV infection detected by DNA testing occurs in the late teen-early 20's age group with estimates as high as 35% [20, 22], dipping to around 10% in the adult woman with increases in prevalence again seen starting in several populations of women aged 35–55 years with a second peak in prevalence a decade later [20].

Most research on DNA detection of HPV infections has focused on the highest prevalence group of females 16–25 years old attributing the peak rates to sexual activity. In sexually active women older than 25 years, the incidence of oncogenic HPV infections is 5 to 15% [40–42] with less than a fifth of the infections attributed to HPV 16 or 18 [40, 42–44]. Less data detail longitudinal studies of incident high-risk HPV infection in women older than 40 years and its meaning vis à vis CIN 3 and cervical cancer progression. 

Data available to date indicate that there is a real incidence of new high-risk HPV infections that exceeds 10% in women older than 42 years. Over a 7-year study period these incident infections cause new CIN 3+ lesions in over half of the HPV-infected women [45]. An equal proportion of newly detected infections were explained by recent sexual behavior as by past sexual behavior [46]. In women older than 56 years, the proportion of high-risk HPV types other than HPV 16 causing the incident CIN 3+ is much more than for younger women (72% versus 32%), a progressive decrease in CIN 3+ HPV 16 attribution with aging [47]. 

**Figure 1:**
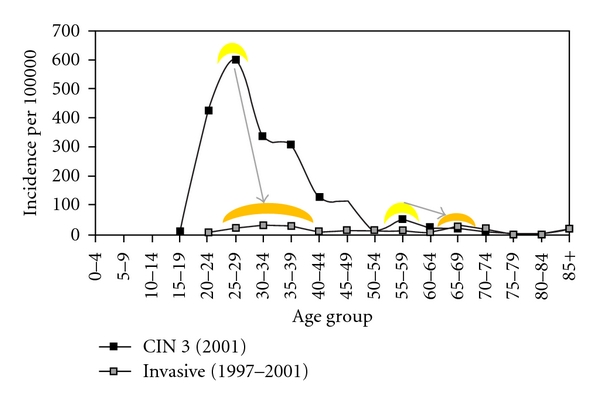
Incidence of cervical intraepithelial neoplasia grade 3 (CIN 3) and cervical cancer in Iceland. Bimodal peaks of CIN 3 incidence (yellow highlighted) occur at 25–29 years of age and again at 55–59 years of age while the bimodal peaks of cervical cancer incidence (orange highlighted) are offset to five to ten years later in the 30–34-year-olds and the 65–69-year-olds [48].

**Figure 2:**
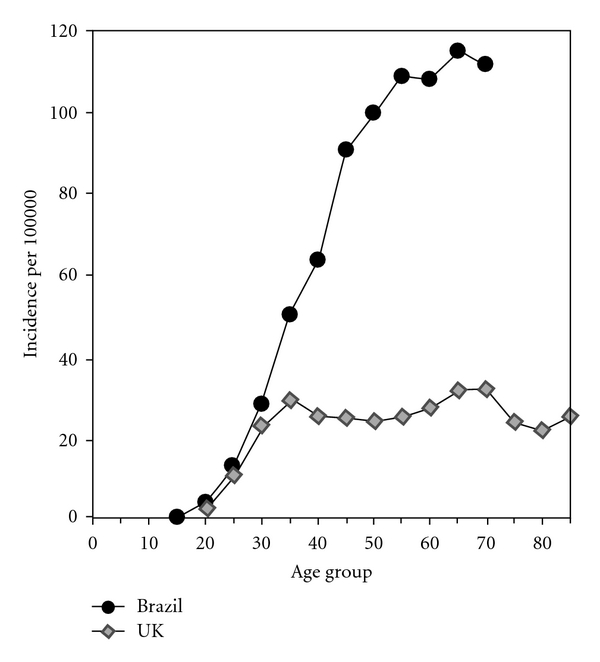
Age-specific incidence of cervical cancer in countries with and without screening programs. The Brazil data represent countries without a cervical cancer cytology screening program. The UK data support the bimodal distribution of cervical cancers peaking at 35 and 65 years of age as seen in Figure 1. The Brazil data show continued increases in cancer incidence through 70 years that would be unlikely should there be no further incident HPV infections in older women [48].

While we have no longitudinal data on the progression of incident CIN 3 in women older than 40 years to cervical cancer, the public registries of the Nordic countries where screening is well organized show bimodal distributions of incident CIN 3 and cervical cancer that are separated by the expected 5–10-year time lag (Figure 1). Likewise, in countries without screening programs, the age-specific incidence of cervical cancer continues to increase with age (Figure 2), rather than plateauing or decreasing as would be seen if there was no risk of cervical cancer from new HPV exposure at an older age.

The role of past infections as a source of latent viral reactivation for the older woman has been seriously challenged by a recent analysis of a ten-year cohort of nearly 2500 women [49]. In this study, women older than 40 years had a similar or higher rate of high-risk HPV reinfection from new partners compared to the initial infection rate for the same HPV types at younger ages. These data suggest that a woman is not fully protected by a first HPV infection earlier in life because she is at a similar risk of acquiring both different high-risk HPV types and the same HPV type she previously had seemed to clear [49]. The cause of the new infections in women older than 40 years was correlated with having new sexual partners only, not from past infections, negating the role of latent viral reactivation. In addition, viral loads of new infections in the previously infected women were comparable to the viral loads of initial infections suggesting that natural immunity also played no protective role in reinfection with the same or different HPV types [49].

### 1.6. Continued Lifetime Risk of Cancer

Among women with a high-risk HPV infection that progresses to a CIN 3 lesion there is an increased risk of other anogenital cancers beyond the cervix. Women have a 3–12-fold increased chance of developing another anogenital cancer (vagina, vulva, anus) within 10 years with increasing risk in the second decade posttreatment for their CIN 3 lesion [50–52]. In addition, cervical cancers can recur despite highly effective excisional treatments for CIN 3 [53] which is only partially explained by incomplete excisions [54].

### 1.7. Distribution of HPV Types Changes according to the Natural History Stage

The distribution of high-risk HPV types varies among increasingly severe cytologic changes and cancer. The prevalence of HPV 16 and 18, the HPV types of most oncogenic potential, is very low among the general population. In the USA population, HPV 16 is the sixth most prevalent infection at 1.5% with HPV 18 occurring in 0.8% of the population [55]. Globally, the prevalence in a normal population is about the same, 2–4%, for HPV 16 and 18 [20]. Even among the highest risk group of women, the baseline prevalence of HPV 16 and 18 DNA positivity maximizes at 9% and 4%, respectively [28, 47, 56, 57]. 

In contrast, the prevalence of HPV 16 and 18 increases to 27% in women with low-grade cytologic abnormalities (low-grade squamous intraepithelial lesions (LSILs)) which by definition means infection with HPV. In women with high-grade squamous intraepithelial lesions (HSILs), the prevalence of 16 and 18 increases to about 50%. Finally, 67% of the squamous cell carcinomas and 75% of adenocarcinomas are caused by HPV 16 and 18 (Table 1) [58–62]. 

**Figure 3:**
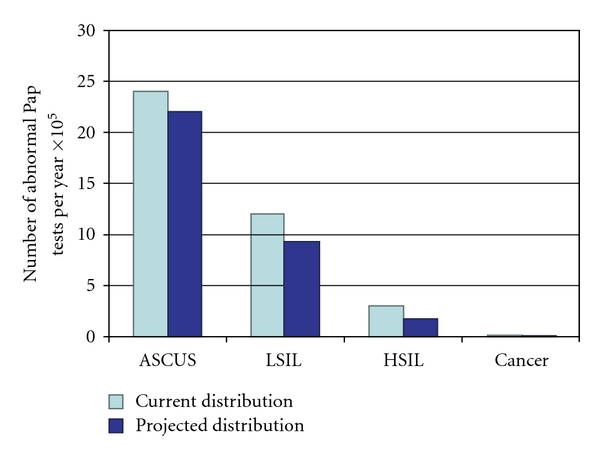
HPV 16 and 18 prevention will reduce cytologic abnormalities by small amounts [73]. In a screened population, while the greatest prophylactic vaccine protection will be in preventing over 70% of cervical cancers, the absolute numbers of cancers available to prevent are quite small. On the other hand, the estimated 8% reduction in ASCUS and 23% reduction in LSIL will yield a much larger absolute number of women who are prevented from further medical work-up [73]. It is this prevention of further medical follow-up that drives the cost-effectiveness of prophylactic vaccination in screened populations [73].

Prophylactic HPV vaccination will have little cancer-reducing effect in the general screened population, but may prevent cervical cancer among those with no screening opportunities. Prophylactic vaccination may help decrease the numbers of women who develop abnormal cytology screens among those who are screened. Modeling indicates that HPV vaccination will prevent potentially 17% of the abnormal Pap tests based on current knowledge of HPV type distribution (Figure 3) [73] and only a very few cancers that Pap testing would not have detected [74, 75], not enough to lower the population incidence of cervical cancer lower than what screening already accomplishes [76].

### 1.8. Modeled Economic Policy Implications

Cervical cancer is the most prevalent in absolute numbers and absolute costs of all the HPV-associated diseases. It comprises 88% of the worldwide burden of HPV-associated cancers and consumes 92% of the monies spent on HPV-associated diseases [77–82]. Cervical cancer represents 6.8% of all female cancers, and 3.2% of all cancers globally [77]. Prevention of CIN 2+ and cervical cancer is the driving force for Cervarix use. 

Four times as many cervical cancers occur in countries without quality-assured screening programs as in those countries with ongoing organized screening programs [77]. The incidence of cervical cancer in countries without screening ranges between 50 and 80/100,000 women [83, 84]. Cervarix will have the highest impact in these countries if given to young women prior to HPV exposure, which public health authorities have targeted as the sexually naive 11-12-year-old age range. Under favorable assumptions, Cervarix could achieve a cervical cancer incidence as low as 9.5/100,000. If the duration of vaccine efficacy is less than 15 years, though, cost-effectiveness analyses show that shifting the recommended age of vaccination to 15 years becomes a much more affordable option [76, 85]. 

Cervical cancer incidence in countries with well-established cytology screening programs ranges from 4 to 10/100,000 women [83], mostly below the incidence that Cervarix can achieve. Yet, among those screened, the incidence of CIN 3 ranges from 1.6 to 16 times the incidence of cervical cancer [48]. In the USA the average incidence of CIN 3 is 150/100,000 women per year with a peak incidence around 800/100,000 women per year in the 25–29-year age group [86]. The incidence of abnormal cytology screens is estimated at 7800/100,000 women per year, another magnitude increase [23]. While public health authorities still target the sexually naive 11-12-year-olds in countries with screening, the benefit will not be reduction of cervical cancer incidence, but instead, a reduction in abnormal cytology screens and incidence of CIN 3 [74]. This benefit is realized within four years of vaccination of sexually active women [57] and is not dependent on the duration of vaccine efficacy exceeding the 15-year threshold or on the coverage of females or males vaccinated as documented in economic models [76, 79, 85]. It is only by continued cytology screening, regardless of the option of vaccination, that the low incidence of cervical cancer can be maintained [74, 87].

## 2. Overview of the Market

Cervarix will be most effective in countries without quality-assured cervical cytology screening programs. In these countries, Cervarix may reduce the incidence of cervical cancer to 9.5/100,000 women if the duration of efficacy exceeds at least 15 years [76]. Modelers agree that duration of vaccine efficacy is the primary parameter determining cost-effectiveness of vaccination followed closely by the cost of the vaccine [74, 76, 80–82, 88–100]. Given the very high one-dose efficacy seen in preliminary trials for Cervarix, the possibility of reducing programmatic implementation costs as well as vaccine costs makes Cervarix an attractive public health vaccine. The competitor HPV vaccine, Gardasil, can reduce the incidence of cervical cancer, under best assumptions, to 14/100,000 in three doses [101]. 

Where cervical cancer screening programs are routinely followed, the option to vaccinate with Cervarix may result in a reduction of abnormal cytologies: in the USA estimates are a reduction to 3400/100,000 women per year and CIN 3 lesions to 11/100,000 women per year. This outcome is clinically quite valuable as it reduces the psychological and reproductive morbidity associated with the screening process [102]. The competitor HPV vaccine, Gardasil, may also effectively reduce abnormal cytology screening rates to 4200/100,000 women per year and CIN 3 lesions to 42/100,000 women per year [101]. 

Quality assured cytology screening programs are still the main deterrent and preventer of *cervical cancer* in industrialized countries, though, not vaccination [74, 76]. The introduction of Cervarix in well-screened nations has the potential to worsen the incidence of cervical cancer if the population participation in the routine cytology screening drops below 70% [74], as has already been observed [75, 103]. Until we have data to show that the duration of vaccine efficacy exceeds the threshold for public utility, ongoing screening programs should not be altered. 

An individual, not population-based or publicly funded, benefit of Cervarix also includes vaccinating seropositive/DNA-negative women (women already exposed but currently not expressing infection) and older women up to 55 years of age; these individual uses of Cervarix are highly valued clinical options based on published study data, not current public health targets. The competitor HPV vaccine, Gardasil, does not offer these benefits [101].

## 3. Composition

Cervarix contains 20 micrograms each of HPV 16 and 18 L1 capsid proteins, called virus-like particles (VLPs). VLPs are composed of self-assembling L1 major capsid proteins. Accessibility of the conformational epitopes for antigen presentation is maximized by the adjuvant, which in turn has contributed to the cross-protection to three phylogenetically related HPV types of both HPV 16 and 18.

The AS04 adjuvant was developed to mimic the Toll-like receptor 4 agonist. This enhancement increases the local cytokine response which results in direct stimulation of antigen-presenting cells and subsequent cellular and humoral immune responses which ultimately effect stronger and more enduring antibody responses [104]. AS04 contains 500 micrograms of aluminum hydroxide and 50 micrograms of 3-O-desacyl-4′-monophosphoryl lipid A (MPL) which is a lipopolysaccharide of *Salmonella minnesota* R595 [105]. AS04 has been found safe and tolerable in trials and in post-marketing surveillance of a hepatitis B vaccine adjuvanted with AS04 (Fendrix) used in both those with and without an impaired immune system [63, 106, 107]. The AS04 adjuvant system has consistently provided higher and longer antibody titers compared to aluminum salt adjuvants both in animal models and in human studies [63, 106–108]. 

There are additional manufacturing components used in the Baculovirus expression system of *Trichoplusia ni* insect cells, to result in the final 0.5 mL dose of Cervarix (Table 2) [109]. Long-term stability of the vaccine is attributed to the L1 VLP manufacturing process and to the AS04 adjuvant [105, 110].

## 4. Efficacy

Cervical cancer is the prevention goal. As it is not ethical to allow women in a placebo arm of a trial to develop cervical cancer when screening and treatment for CIN 2/3 and adenocarcinoma in situ (AIS) lesions is standard of care, the most closely related surrogate endpoint to cervical cancer that can be studied for prevention in clinical trials is CIN 2/3 and AIS. As the mechanism of vaccine action is to prevent HPV infection, not the development of CIN 2/3/AIS after an infection, a second surrogate endpoint for prevention is incident and persistent HPV infection [111]. 

Cervarix has documented high efficacy against the combination endpoint of cancer precursors CIN 2/3, AIS, and invasive cervical cancer (CIN 2+) caused by HPV 16/18 (Table 3) [57, 64, 65]. This efficacy lasts for at least 9.4 years. 

### 4.1. Additional Efficacy against HPV 31, 33, and 45

In addition, Cervarix provides protection in HPV naive women from both six- and twelve-month persistent infection, and CIN 2+ caused by HPV 31, 33, and 45 lasting at least 8 years (Table 4) [112]. This additional protection is significant as it proves cross-protection for both HPV-16 and HPV-18 phylogenetically related types. The HPV-16-related types (31, 33, 52, and 58) are more commonly associated with squamous cell carcinomas, whereas the HPV-18-related types (45 most dominantly) are associated with adenocarcinomas. Together HPV 45, 31, and 33 collectively account for about 15% of invasive cervical cancers, and nearly 20% of all adeno- or adenosquamous carcinomas, with HPV 45 being the dominant contributor after HPV 16/18 [60]. 

Extrapolating from HPV distribution studies and the cross-protection efficacies, Cervarix is expected to protect against more than 90% of adenocarcinomas, those cancers that occur in younger women, are most difficult to detect by screening programs, present at later-stages, and thus have higher mortality [60, 61, 66, 113]. In addition Cervarix is expected to prevent more than 80% of squamous cell carcinomas for a combined effect of preventing over 85% of all types of cervical cancers. This substantial impact at the cancer level is supported by trial data at the CIN 2+ level indicating a 70% efficacy against all CIN 2+ lesions regardless of HPV type causation, which by epidemiologic studies is expected to be only 50% (Table 4) [59]. End of study analyses indicate 93% efficacy against all CIN 3+ lesions regardless of HPV type causation [114].

### 4.2. Prevention of Abnormal Cytology, CIN 2+, and Excisional Treatments

Among women who received Cervarix when seronegative and DNA-negative for HPV 16/18 and who participated in repeated cytology screening, Cervarix prevented more than 20% of all abnormal cytology in the first 4 years after vaccination (Table 5) [57]. Cervarix prevented 26% of the clinically indicated colposcopy exams and reduced the rate of excisional procedures for precancerous disease by nearly 70% [57].

### 4.3. Efficacy in Seropositive Females

Cervarix has excellent efficacy against CIN 2+ caused by HPV 16/18 in women who are already seropositive for the same HPV type, but DNA-negative at the time of initial vaccination (Table 6) [67]. As the meaning of serostatus defined by the current assay methods for both vaccines has been recently questioned [115], the data on vaccine efficacy in the set of vaccinated subjects without current HPV 16/18 infection (DNA-negative for HPV 16/18) at baseline regardless of serostatus becomes important. The vaccine efficacy against CIN 2+ caused by HPV 16/18 regardless of serostatus among all vaccinated women HPV-DNA-negative for HPV 16/18 at baseline is 99% to 92% with tight 96.1% confidence intervals of 84–97 [67]. This efficacy is reassuring because population data indicate that nearly 10% of the 11-12-year-old target age group has already seroconverted for prior exposure to high-risk HPV types, potentially due to vertical or horizontal mother to child transmission [38, 116]. This high efficacy is supported by its corresponding strong immunologic data.

### 4.4. Efficacy in Less Than 3 Doses

Vaccine efficacy is currently defined after three doses are administered. USA and European data show that less than 20% of 13-year-old girls have received all three doses of vaccine [117, 118] with anecdotal evidence indicating that only a fraction of those receiving three doses received all three doses within the one year time span for the expected efficacy to ensue. Catch-up vaccination in the 19–26 year-old women is also less than 20% [119], a critically cost-effective age group of females if vaccine efficacy is less than 10 years [81]. 

Early data show that Cervarix remains 100% efficacious with just one-dose (Table 7) [68]. While this must be proven for regulatory approval, this property of Cervarix both simplifies implementation and lessens cost for cervical cancer prevention everywhere.

### 4.5. Efficacy in Older Women by Immunobridging

Cervarix studies in women through 55 years have been ongoing for more than four years with immunogenicity results very similar to the 10–15-year-old and the 15–26-year-old population thus bridging to a similar efficacy for the older aged cohort (Table 8) [69–72, 120]. While this indication has received regulatory approval in 60 countries, the option to vaccinate a woman older than 26 years appears to be a promising clinical option for individual women in all countries.

### 4.6. No Therapeutic Efficacy

While Cervarix provides very high efficacy regardless of serostatus, Cervarix does not cause regression of HPV DNA 16/18 infections already present, nor does Cervarix accelerate progression to CIN 3+ from the current infection [121].

## 5. Immunogenicity

As discussed, cost-effectiveness models indicate no cancer prevention occurs if duration of efficacy is too short. The minimum time analyzed is 10 years of efficacy, most use 15- or 20-year durations; if less than this, the cancers are merely postponed, not prevented [76, 79–81]. While Cervarix trials continue to accrue efficacy years beyond the documented 9.4 years, the supplementary and supportive traditional surrogates of efficacy are immunologic responses, despite not knowing the full clinical ramifications of their meaning, nor the degree of influence that smoking or hormonal contraception may cause.

### 5.1. In-Vitro Head-to-Head Trials between Cervarix and Gardasil

T helper cells are important for several immune functions. These include B-cell differentiation, sustained memory, activation of recall reactions, and thus anamnestic responses [122]. T helper cell (CD4+) frequencies at 18 months after first vaccination were a robust 93% for HPV 16 and 79% for HPV 18, significantly higher than the competitor vaccine in head-to-head trials [101, 123]; similarly, Cervarix had superior frequencies of T cells for HPV 31 and 45 than the competitor vaccine at one month after the third vaccine dose [122]. HPV 6 and HPV 11 L1-reactive T cells were induced after Cervarix administration at similar frequencies as seen in recipients of Gardasil [122]. Protection against genital warts from HPV 6/11 has been documented in the Cervarix vaccinated population in the UK [124].

At 18 months, Cervarix-induced a robust memory B cell response for HPV 16 in 87% of subjects and for HPV 18 in 75% of subjects, both significantly superior to Gardasil [101, 123].

#### 5.1.1. Systemic Antibody Titers in Head-to-Head Trials between Cervarix and Gardasil

**Figure 4:**
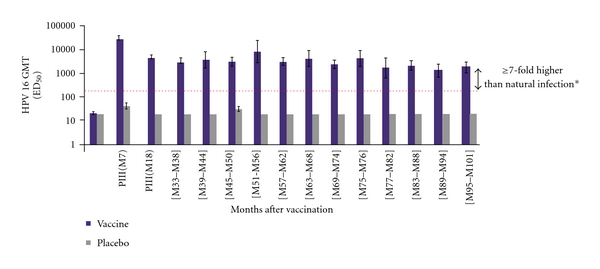
Sustained anti-HPV-16 titers continue through 9.4 years among ATP cohort for immunogenicity [64, 65]. GMT means geometric mean titres for anti-HPV-16 neutralising antibodies by pseudovirion-based neutralisation assay (PBNA). *Horizontal line represents the neutralising antibody level in women from a phase III efficacy study who had cleared a natural infection before enrollment. ATP cohort for immunogenicity means women who met all eligibility criteria, were seronegative at baseline for HPV 16/18 and DNA-negative for 14 oncogenic HPV types at baseline, normal cytology, complied with study procedures in preceding and current studies, and had data available for at least one vaccine antibody blood sample. PRE means prevaccination.

**Figure 5:**
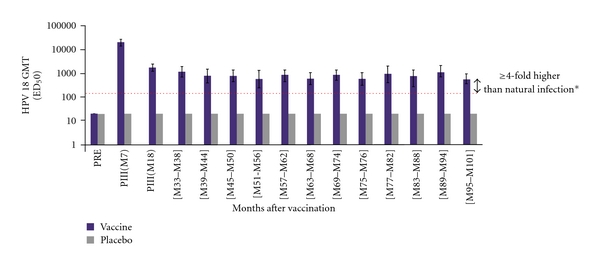
Sustained anti-HPV-18 titers continue through 9.4 years among ATP cohort for immunogenicity [64, 65]. GMT means geometric mean titres for anti-HPV-18 neutralising antibodies by pseudovirion-based neutralisation assay (PBNA). *Horizontal line represents the neutralising antibody level in women from a phase III efficacy study who had cleared a natural infection before enrollment. ATP cohort for immunogenicity means women who met all eligibility criteria, were seronegative at baseline for HPV 16/18 and DNA-negative for 14 oncogenic HPV types at baseline, showed normal cytology, complied with study procedures in preceding and current studies, and had data available for at least one vaccine antibody blood sample. PRE means prevaccination.

**Figure 6:**
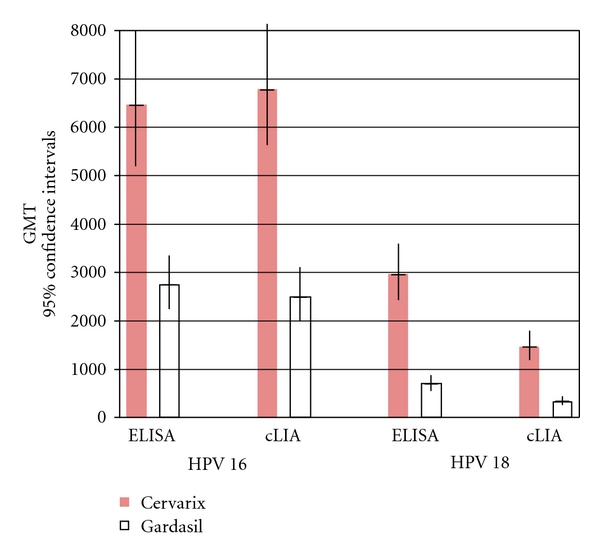
Comparative anti-HPV-16 and anti-HPV-18 titers measured in two assays for Cervarix and Gardasil [126]. Immunogenicity comparisons between Cervarix and Gardasil one month after 3rd dose (month 7) in 18–45-year-old women: ELISA versus cLIA measurement systems. ELISA: enzyme-linked immunosorbent assay in ELISA units/mL (EU/mL). cLIA: competitive Luminex immunoassay in milliMerck units/mL (mMerckU/mL). GMT means geometric mean titer.

Systemic antibody responses induced by Cervarix, measured by the pseudovirion-based neutralisation assay (PBNA), were robust, despite protective serologic antibody titers having not been defined. Cervarix maintained a 100% seropositivity for both HPV types with substantial titers above natural infection for the full 9.4 years (Figures 4 and 5) [125]. When measured in a head-to-head trial with Gardasil one month after three doses of vaccine, Cervarix-induced 4-fold more anti-HPV 16 titers and 7-fold more anti-HPV-18 titers than Gardasil, and at 18 months Cervarix-induced antibodies were still 2.5-fold higher for HPV 16 and 5-fold higher for HPV-18 than those induced by Gardasil in 18–45-year-old women [101, 123]. Head to head trial antibody measurements in different assay systems for Cervarix and Gardasil showed many-fold higher response by Cervarix than by Gardasil putting an end to the argument that the difference in antibody titers is caused by the antibody measurement systems (Figure 6) [126].

#### 5.1.2. Local Antibody Titers in Head-to-Head Trials between Cervarix and Gardasil

Local antibody titers at the cervical squamocolumnar junction within the cervicovaginal mucous may play an important adjuvant role in the prevention of HPV 16/18 infection [127]. The value of mucosal antibodies is vigorously debated ranging from no proven value in human biologic systems to time-enhanced virion neutralization because of immediate antibody presence. Antibodies transudate from the dermal capillary network to place antibodies directly at the squamocolumnar junction where basal cell invasion, is most vulnerable [128]. Eighteen months after vaccination Cervarix-induced cervicovaginal mucous antibodies to HPV 16 were measurable in 21% of women and to HPV 18 in 7% of women. This was significantly higher than the 14% and 0% in Gardasil recipients at the same time point, respectively, in head-to-head trials [123].

### 5.2. Immunogenicity in Females with Prior Exposure

**Figure 7:**
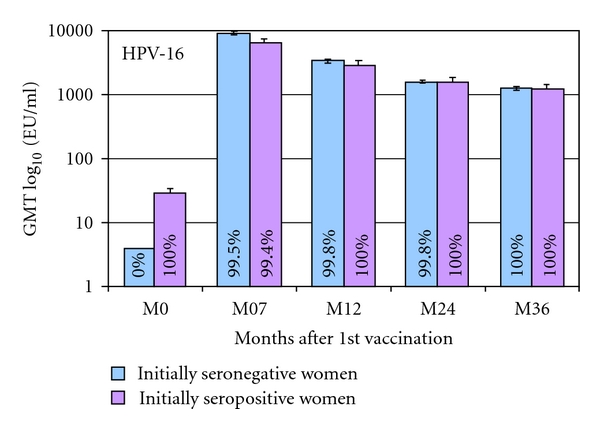
Geometric mean titers and seropositivity rates measured by ELISA for anti-HPV-16, by serostatus in women 16–26 years old [129]. ATP cohort for immunogenicity. Bars show log_10_ geometric mean titer and 95% confidence interval. % = proportion of women seropositive for antigen. For the GMT calculation, seronegative women were assigned a value of half the assay cutoff level. The number of women with evaluable blood sample shows the number of women who were seropositive or seronegative at baseline. ATP: according to protocol; HPV: human papillomavirus; GMT: geometric mean titer.

**Figure 8:**
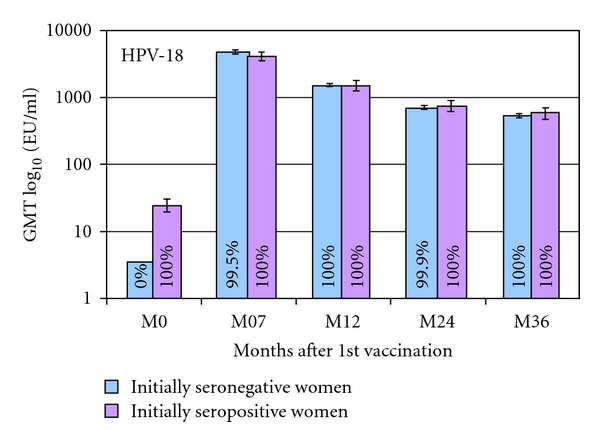
Geometric mean titers and seropositivity rates measured by ELISA for anti-HPV-18, by serostatus in women 16–26 years [129]. ATP cohort for immunogenicity. Bars show log_10_ geometric mean titer and 95% confidence interval. % = proportion of women seropositive for antigen. For the GMT calculation, seronegative women were assigned a value of half the assay cut-off level. The number of women with evaluable blood sample shows the number of women who were seropositive or seronegative at baseline. ATP: according to protocol; HPV: human papillomavirus; GMT: geometric mean titer.

Cervarix induces equally high anti-HPV 16 and anti-HPV-18 titers in HPV-DNA-negative women regardless of their serostatus (Figures 7 and 8) [129, 130]. This supports the efficacy data from seropositive/DNA-negative women. This data should provide reassurance to physicians and women that vaccination with Cervarix after HPV exposure will offer the same high immunological protection as in vaccinated seronegative/DNA-negative women.

### 5.3. Immunogenicity of Women Aging 25–55 Years

**Figure 9:**
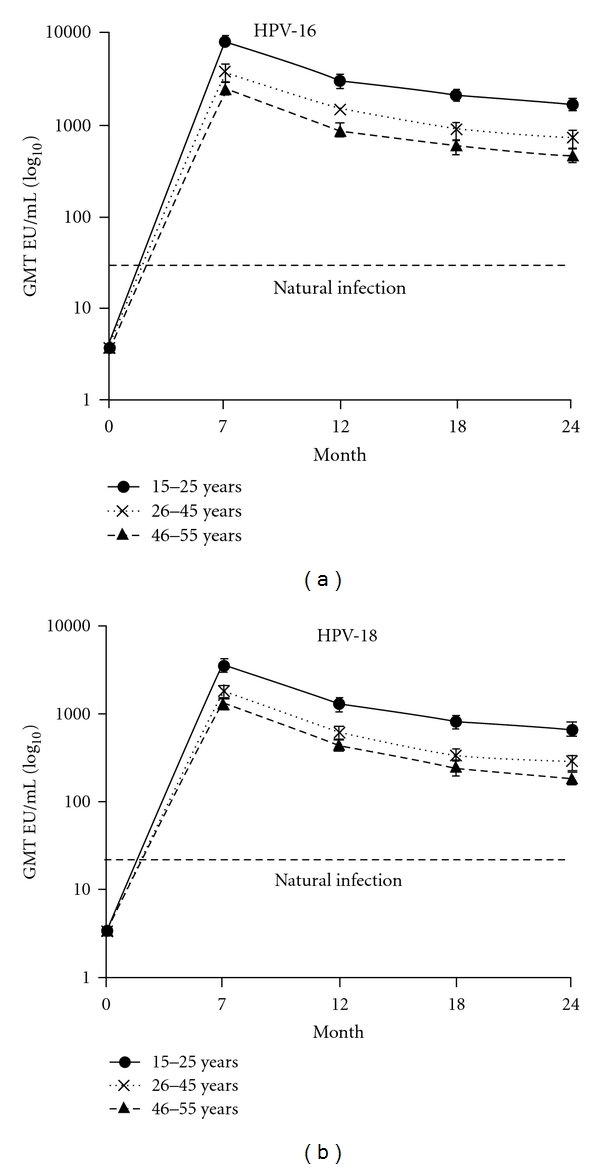
Antibody titers in women 15–55 years of age seronegative at baseline in the ATP cohort over time [71]. GMT means geometric mean titre and is shown with 95% confidence intervals; EU/mL = ELISA units per milliliter. Arrows indicate the vaccination time points (months 0, 1, and 6). Seropositivity is defined as ≥8 EU/mL for anti- HPV-16 and ≥7 EU/mL for anti-HPV-18. Natural infection is from the GMTs in women seropositive DNA-negative for HPV-16 and HPV-18 from the PATRICIA trial [129].

Table 8 and Figure 9 indicate that even the 46–55-year-old women mount a peak antibody response to Cervarix that is at least 50 fold higher than natural infection induces, and this response stays many-fold higher than natural infection titers through at least month 48 for both HPV 16 and HPV 18 [71, 131].

### 5.4. Immunogenicity of Less than Three Doses of Cervarix

In 9–25-year-old females Cervarix induces the same peak antibody titers for HPV-16 and 18, measured at month 7 after first injection for three doses (given at 0, 1, and 6-months) as for two-doses (given at 0 and 6-months). The two dose regimen contained double the antigen concentration at the 6-month dose [135]. 

### 5.5. Immunogenicity in Males

**Figure 10:**
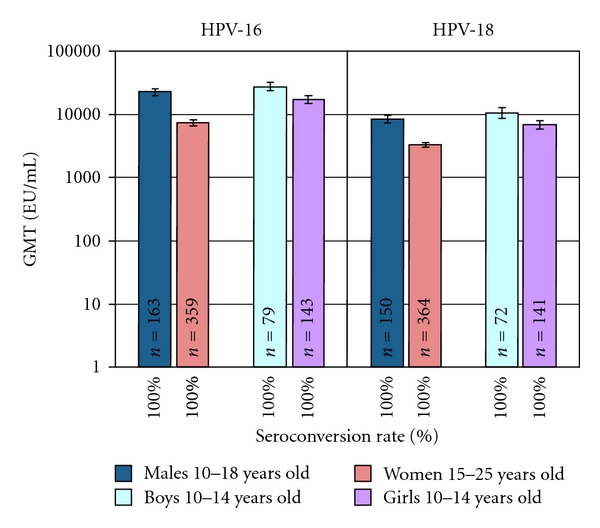
Seroconversion and antibody response to HPV 16/18 in males compared to females [132]. GMT means geometric mean titer measured at month 7, one month after the third dose of Cervarix. All subjects were seronegative at study entry. Data based on ATP immunogenicity cohort [132]. ATP means according to protocol.

With the opportunity to prevent other HPV-associated cancers in males, Cervarix has been trialed in males to document its induced immunologic responses. Among males 10–18 years old, Cervarix induces higher peak antibody titers than in females of similar ages. Specifically, after three doses in males 10–14 years of age anti-HPV 16 and anti-HPV-18 titers are higher than those induced in 10–14-year-old females (Figure 10) [132]. Titers are sufficiently robust to support immunobridging to efficacy in males.

### 5.6. Coadministration with Other Vaccines

**Figure 11:**
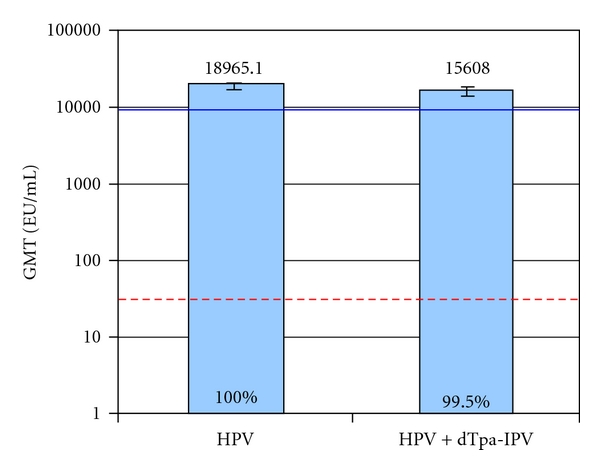
Peak anti-HPV 16 titers with Cervarix compared to Cervarix co-administered with a pentavalent vaccine in seronegative females 10–18 years old at study entry [133]. Anti-HPV 16 titers were measured one month after the third dose of Cervarix in the according to protocol immunogenicity cohort of study subjects. Anti HPV 16 seroconversion is equivalent in the two groups studied, represented by the percentages at the base of the columns. The blue line represents the anti-HPV 16 response in women 16–26 years old, and the red dotted line represents the anti-HPV 16 titers in women with natural infection who had cleared their infection (natural infection titers). GMT means geometric mean titer shown with 95% confidence interval bars. EU means ELISA unit. HPV means human papillomavirus vaccine, Cervarix, administered at month 0, 1 and 6. HPV+dTpa-IPV means Cervarix co-administered with diphtheria, tetanus, acellular pertussis and inactive polio vaccine at month 0, and Cervarix alone administered at months 1 and 6.

**Figure 12:**
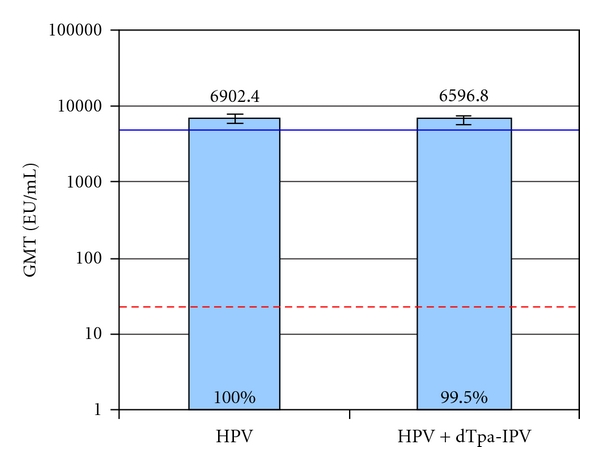
Peak anti-HPV-18 titers with Cervarix compared to Cervarix co-administered with a pentavalent vaccine in seronegative females 10–18 years old at study entry [133]. Anti-HPV 18 titers were measured one month after the third dose of Cervarix in the according to protocol immunogenicity cohort of study subjects. Anti HPV 18 seroconversion is equivalent in the two groups studied, represented by the percentages at the base of the columns. The blue line represents the anti-HPV-18 response in women 16–26 years old, and the red dotted line represents the anti-HPV-18 titers in women with natural infection who had cleared their infection (natural infection titers). GMT means geometric mean titer shown with 95% confidence interval bars. EU means ELISA unit. HPV means human papillomavirus vaccine, Cervarix, administered at month 0, 1 and 6. HPV+dTpa-IPV means Cervarix co-administered with diphtheria, tetanus, acellular pertussis and inactivated polio vaccine at month 0,and Cervarix alone administered at months 1 and 6.

The adolescent platform of vaccination was designed around the revised recommendations of HPV, pertussis, and meningococcal vaccination at 11-12 years of age [136]. Coadministration of vaccines at this age requires data to show both equivalent immunogenicity and safety as would have been obtained without co-administration. Cervarix was trialed with a combined reduced-antigen content diphtheria-tetanus acellular pertussis—inactivated poliovirus vaccine (dTpa-IPV, Boostrix) in females 10–18 years old seronegative for HPV 16/18 at baseline [133]. The trial showed that titers one month after the third dose of Cervarix when administered alone were the same as the titers induced from Cervarix with Boostrix; these anti-HPV16 and anti-HPV-18 titers are hundreds of fold higher than natural infection titers (Figures 11 and 12). Three ongoing trials pair Cervarix with TDaP (Boostrix) and/or meningococcal conjugate vaccine (Menactra), Cervarix with hepatitis B vaccine (Engerix B), and Cervarix with a combination of hepatitis A and B vaccines (Twinrix) (Clinical Trials registration numbers NCT00369824, NCT00652938, and NCT00578227, resp.) [137]. These represent the vaccines currently recommended in the adolescent platform in the United States.

## 6. Safety

Safety has been discussed in detail in previous publications [130, 138]. In general, Cervarix is safe for most women: adolescent girls, younger women, and older women through 72 years of age [57, 69–72, 129, 139]. Local side effects of pain, erythema, and induration occur after vaccination with resolution within 2-3 days. Phase II and phase III randomized controlled trials (PATRICIA trials) did not reveal any serious adverse events occurring more often in the vaccinated group than the placebo group. After 9.4 years of follow-up, new onset chronic disease did not occur any more frequently in the vaccine than placebo group. Teratogenic and pregnancy related side effects have not been identified with Cervarix [140, 141], but post-marketing surveillance will continue to monitor the rate of spontaneous abortion among Cervarix recipients whose pregnancies occurred around the time of vaccination [142]. 

Co-administered vaccines did not produce worse local injection side effects and did not increase the reporting of serious adverse events; there was detailed follow-up for 30 days after vaccination and for those subjects who became pregnant [133].

## 7. Post Marketing Surveillance

Post-marketing surveillance continues to follow specific pregnancy related side effects. In addition the FDA sponsors the general Vaccine Adverse Events Reporting System (VAERS) to periodically reassess vaccine safety, making changes in recommendations only if the incidence of adverse events is more than twice the very rare incidence of 1/10,000 women [143]. To date there are only 39 reports in VAERS after Cervarix administration, 97% of which are non-serious report dyspnea and fainting [144]. Fainting has been a recognized outcome after vaccination, especially in the adolescent population, which has led to the recommendation of 15 minutes of observation after any HPV vaccination [109, 145]. There has been one publication of autoimmune demyelinating neurologic disease that completely resolved within 3 weeks without sequelae after the second dose of Cervarix [134].

## 8. Regulatory Affairs

Cervarix was approved by the USA Food and Drug Administration (FDA) in October 2009. It is currently licensed in over 100 countries worldwide with over 60 countries approving Cervarix for women older than 25 years. The vaccine is approved for use in the USA in females aged 10 years through 25 years for the prevention of HPV 16/18 attributed cervical cancer, CIN 2+, adenocarcinoma in situ, and CIN 1+ disease [146]. The regulatory approval acknowledges cross-protection to HPV types beyond HPV 16/18. 

The USA based Advisory Committee on Immunization Practices (ACIP) recommends routine vaccination of females aged 11 or 12 years with 3 doses; the vaccination series can be started as early as 9 years. Likewise, vaccination is recommended for females aged 13 through 26 years who have not been vaccinated previously or who have not completed a 3-dose series of HPV vaccination. If a female reaches the age of 26 years before the vaccination series is complete, remaining doses can be administered after the age of 26 years. The second dose should be administered one to two months after the first dose. The minimum interval between the first and second dose of vaccine is 4 weeks and between the second and third dose is 12 weeks. The minimum interval between the first and third dose is 24 weeks [145]. 

The product insert recommends Cervarix to be administered intramuscularly in three separate shots, with the initial 0.5 mL dose being followed by two additional shots at one and six months [109]. 

## 9. Conclusions

**Figure 13:**
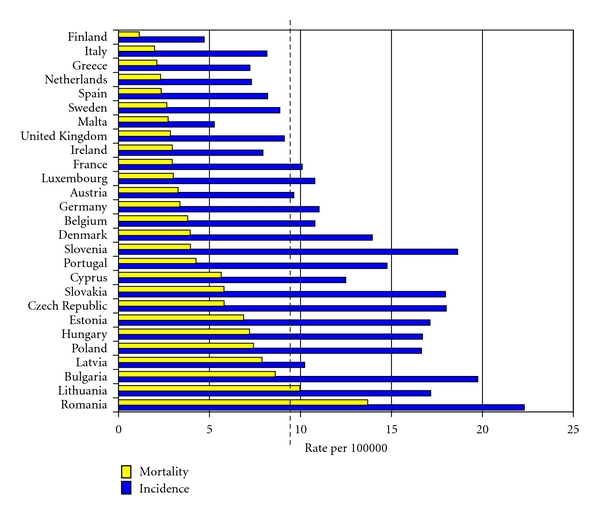
Age standardised mortality from and incidence of cervical cancer in the 27 Member States of the EU (2004) [134]. Dotted line represents the estimated incidence of cervical cancer that Cervarix can achieve at 9.5/100,000 women. Countries with incidences (blue bar) to the right of the dotted line may have a population benefit reduction in cervical cancer incidence.

Cervarix has the potential to reduce the burden of cervical cancer in either one or three doses in those females who are seronegative or seropositive for HPV 16/18. In populations without organized cervical cancer screening, Cervarix can lower the incidence of cervical cancer from the current 50–80/100,000 women to 9.5/100,000 women. In Europe, 18 of the 27 member states of the European Union have cervical cancer incidences above 9.5/100,000 (Figure 13) [147]. 

In countries with cervical cancer screening programs, Cervarix has the potential to reduce the population incidence of cervical cancer in those subpopulations who do not participate regularly in the screening programs. In the USA this would be the Hispanic and Black populations with current cervical cancer incidences of 12.8 and 11.1 per 100,000, respectively [148]. 

The largest population benefit of Cervarix in the screened population, though, is the reduction of abnormal Pap test results, colposcopies, and excisional treatments. reduction in CIN 3+ regardless of HPV causation is shown to be 93% for those vaccinated between 16–26 years [114]. In addition, the benefit of Cervarix in women older than 25 years is to reduce the incidence of CIN 2+, currently equivalent to the incidence of all other HPV -associated cancers in women combined (anal, vaginal, vulvar, and oropharyngeal). Cervarix has the greatest potential, more than any known vaccine or screening method to reduce the burden of cervical adenocarcinomas in both the screened and unscreened populations by over 90% [60, 66]. 

Cervarix induces anti-HPV 16 and anti-HPV-18 titers in males that exceed titers in similarly aged females, and Cervarix has a proven duration of efficacy for at least 9.4 years.

## 10. Expert Commentary

Cervical cancer screening programs have been very successful. Evidence now confirms that decreases in population participation in screening programs do cause an increased overall population incidence of cervical cancer [75, 103]. This increase in cervical cancer is also predicted from cost-effectiveness models which show that high screening participation is more important than population vaccination for minimizing cervical cancer incidence [74]. Cancer incidence will also increase if vaccine efficacy is time limited and those vaccinated neglect participating in routine screening [74, 76, 85]. 

Should vaccination be an option that women choose for their cervical cancer protection, Cervarix is an excellent choice for both screened and unscreened populations due to its long-lasting protection, its broad protection for at least five oncogenic HPV types, the potential to use only one-dose for the same level of protection, and its safety. 

The most efficient use of Cervarix is in countries without any cervical cancer screening programs where the incidence of cervical cancer can be reduced to 9.5/100,000 women by vaccinating young females. This is the public health target of the World Health Organization (WHO) and most public health authorities. In addition, Cervarix offers the best chance of preventing adenocarcinomas of the cervix. 

Other options, not public health targets at this time, but supported by trial data, include vaccinating women at any age who are not currently infected with HPV 16/18; this is about 98% of women [20, 55]. A major objection to HPV vaccination is the pre-pubescent young age at which parents are asked to make the decision to vaccinate their daughters [149]. Public health organizations target this age group because the data from the vaccine trials show excellent efficacy in females who have not yet been exposed to HPV: both seronegative and DNA-negative. But the Cervarix trial data also show excellent efficacy in DNA-HPV-16/18 negative women regardless of serostatus at vaccination. This is reassuring for the youngest age vaccinees as epidemiologic data show that 10% are already seropositive to high-risk HPV types at 11-12 years of age. In addition, the efficacy in both seronegative and seropositive females who are HPV-DNA-negative at the time of vaccination is reassuring to females who choose to be vaccinated at an older age. Older age vaccination is strongly supported by data showing that there is little benefit of vaccination at 11-12 years of age if the duration of vaccine efficacy is not at least 15 years [76, 85]. Moreover, much data show that seronegative females may actually have already seroconverted from a prior exposure to HPV but their antibody titers have waned and are now too low to detect. Hence, the conundrum of whether a sexually active woman is truly seronegative becomes moot with high Cervarix efficacy regardless of serostatus. 

Another important individual health benefit, not supported by public health authorities at this time, is the vaccination of older women who are in screening programs [150, 151]. The incidence of CIN 2+ among women older than 45 years is estimated to be 385/100,000 women [47]. While no one knows how quickly incident CIN 2+ in older women will progress to cervical cancer, clinical prudence indicates excisional treatment for this lesion. This incidence is equivalent to the combined incidence of vaginal, vulvar, anal, and oropharyngeal cancers caused by HPV in women of all ages, also 385/100,000 women [152]. If vaccine protection is thought useful for these latter HPV-associated cancers, then it is logical that vaccine protection would also be useful for older women in countries with screening programs, where the true benefit of vaccination is the prevention of new CIN 2+. 

Cervarix is particularly pertinent for the prevention of CIN 2+ in women older than 45 years because 70% of these CIN 2+ are caused by high-risk HPV types other than HPV 16 [47]. The additional high-risk-type protection against HPV 45, 31, and 33 targets the different HPV type distribution seen in precancerous disease of older women. Even women older than 55 years continue to accrue incident oncogenic HPV infections that progress to CIN 3 disease [47]; hence, being able to vaccinate women through 55 years adds individual, if not population, protection. 

Cervarix prevents anal HPV infection in women for HPV 16, 18, 31, 33, and 45 [153], more possible anal cancer prevention than the competitor vaccine, Gardasil.

Finally, while male uses of Cervarix are not recognized by regulatory authorities at this time, should HPV vaccination for male HPV-associated cancers become a health priority, Cervarix has excellent immunogenicity and safety in males.

## 11. Five-Year View

Within five years Cervarix will have completed its extension trial designed to determine long-term immunogenicity, efficacy, and safety past 15 years. Finland has enrolled over 20,000 16-17-year-old women in the PATRICIA trial who are continuing to be followed. Their data will be linked with the Finnish cancer registry and provide CIN 3/cancer incidence rates between the vaccinated and unvaccinated women at 10 years after initial PATRICIA enrollment providing evidence of efficacy past the 15-year threshold cost-effectiveness models have identified [154].

While vaginal and vulvar lesions are a very small proportion of HPV-associated anogenital diseases, and the PATRICIA studies did not include vulvar or vaginal endpoints, the ad hoc collection of VAIN 2+ and VIN 2+ data show vaccine efficacy of 54% in the TVC-N population [155].

Because of the novel adjuvant formulation of Cervarix, this HPV vaccine may have a unique role in protecting those with autoimmune disorders. Trials enrolling women with HIV, juvenile idiopathic arthritis, systemic lupus erythematosus, and juvenile dermatomyositis (NCT00815282) are ongoing and expected to have reportable results within the next five years. 

The development of second-generation vaccines continues as solid global evidence that the high-risk types of most importance include the five types already covered by Cervarix and three additional types (HPV 35, 52, and 58) [60]. Of more importance is the possibility of conjoining the L1 and L2 VLPs to develop a pan-protective vaccine whose one-dose produces at least 15 years of efficacy against all CIN 2+. With this future-generation vaccine, it would be possible to discuss the discontinuation of routine cervical cancer screening programs.

## 12. Key Points

- Cervical cancer prevention, the main purpose of HPV vaccination, is best served by continued participation in Pap screening programs with optional vaccination.
- Cervarix's pertinent strengths are
  - its ability to prevent CIN 2+caused by five oncogenic HPV types for at least 9.4 years [125],
  - its high and sustained T- and B-cell immunologic responses, including serum antibody titers, for HPV-16 and 18 that last at least 8.4 years,
  - its ability to prevent CIN 2+ caused by HPV 16/18 in women DNA-negative for HPV 16/18 but seropositive (already exposed to HPV 16/18) equally well as in seronegative females for at least 8.4 years,
  - its ability to prevent CIN 2+ in one-dose equally well as in three doses for at least 4 years,
  - its ability to immunobridge CIN 2+ efficacy in women through 55 years
  - its ability to prevent about one in five abnormal Pap tests and to prevent nearly 70% of the excisional treatments in women with access to screening.

## Figures and Tables

**Table 1:** HPV genotype distribution among women with increasingly severe cervical abnormalities.

	Normal women [62]	Women with LSIL [58]	Women with HSIL [59]	Women with squamous cell cervical carcinoma [60]	Women with adeno- or adenosquamous carcinoma of the cervix [60, 61]
HPV 16	1.8%	27%	45%	62%	39%–50%
HPV 18	0.7%	9%	7%	8%	32%
HPV 31	0.7%	12%	9%	4%	1%-2%
HPV 33	0.5%	8%	7%	4%	1%–3%
HPV 45	0.5%	5%	2%	5%	12%

**Table 2:** Vaccine composition of a 0.5 mL dose of Cervarix [63].

	Protein subunit component	Concentration	Adjuvant	Manufacturing components	Expression system
Cervarix	HPV 16 L1 VLP	20 *μ*g	AS04: 50 *μ*g of 3-O-desacyl-4′-mono phosphoryl lipid A (MPL) adsorbed onto 500 *μ*g aluminum hydroxide salt	4.4 mg sodium chloride	Baculovirus expression system in *Trichoplusia ni* insect cells
	HPV 18 L1 VLP	20 *μ*g		0.624 mg sodium dihydrogen phosphate dihydrate <40 ng of insect cell and viral protein <150 ng bacterial cell protein	

**Table 3:** Significant efficacies for Cervarix by endpoint, population and time of follow-up.

Endpoint	Population	Vaccine efficacy (96.1% CI)	Average time of follow-up	Reference
6 mo persistent infection caused by HPV 16/18	ATP-E	100% (80, 100)	7.3 yrs	[64]
12 mo persistent infection caused by HPV 16/18	ATP-E	100% (56, 100)	7.3 yrs	[64]
CIN 1+ caused by HPV 16/18	ATP-E	100% (34, 100)	7.3 yrs	[64]
CIN 2+ caused by HPV 16/18	ATP-E	100% (51, 100)	8.4 yrs	[65]
CIN 2+ caused by HPV 16/18	ATP-E*	98% (88, 100)	48 mo	[57]
CIN 3+ caused by any HPV type	TVC-N	93% (79–99)	48 mo	[57]

6-month persistent infection is defined as the detection of DNA from the same HPV type in two consecutive cervical cytology samples collected over any 6-month period and 12-month persistence as detection of the same HPV type in all available cytology samples collected over any 12-month period.

ATP-E means according to protocol for efficacy all women who met eligibility criteria and complied with the protocol, who received three injections, whose baseline Pap was normal, ASCUS or LSIL, and who were seronegative to HPV 16 and 18 and DNA-negative for HPV 16/18 at baseline; cases counting starting one day after the third vaccination.

ATP-E* means those in the ATP-E, but HPV type assignment algorithm was used to resolve causation when multiple HPV types were present.

TVC means total vaccinated cohort: women with at least one injection, seropositive or negative, PCR positive or negative for one or more HPV types at baseline, regardless of Pap result; case counting starting first day after first injection.

TVC-N means TVC-naive: women who received one or more vaccine dose with normal cytology, seronegative for HPV 16/18 and DNA negative for 14 oncogenic HPV types at baseline (16, 18, 31, 33, 35, 39, 45, 51, 52, 56, 58, 59, 66 and 68); case counting starting first day after first injection.

**Table 4:** Cross protection offered by Cervarix [66].

Endpoint	Population	Vaccine efficacy (96.1% CI)	Average time of follow-up
6 month persistent infection	TVC-N		
HPV 31		77% (67, 84)	44 month
HPV 33		43% (19, 60)	44 month
HPV 45		79% (61, 89)	44 month

CIN 2+ associated with	TVC-N		
HPV 31		89% (66, 98)	44 month
HPV 33		82% (53, 95)	44 month
HPV 45		100% (42, 100)	44 month
Regardless of HPV type	TVC-N	93% (79, 99)	44 months

Infection definitions: 6-month definition required the detection of the same HPV type in two consecutive cervical samples, with no negative sample in between, over a minimum of 5 months.

CIN 2+ means cervical intraepithelial neoplasia grades 2 and 3 and adenocarcinoma insitu. Lesions may be coinfected with HPV 16/18.

TVC means total vaccinated cohort: women with at least one injection, seropositive or negative, PCR positive or negative for one or more HPV types at baseline, regardless of Pap result; case counting starting first day after first injection.

TVC-N means TVC naive: women who received ≥1 vaccine dose with normal cytology, seronegative for HPV-16/18 and DNA-negative for 14 oncogenic HPV types at baseline (16, 18, 31. 33, 35, 39, 45, 51, 52, 56, 58, 59, 66, and 68); case counting starting first day after first injection. Subjects were followed for an average 44.3 months after first injection.

**Table 5:** Reduction in abnormal cytology irrespective of HPV causation, colposcopy, and excisional treatments [57].

	Population	Vaccine efficacy (96.1% confidence intervals)
ASCUS	TVC-N	23% (17, 29)
LSIL	TVC-N	24% (14, 33)
HSIL	TVC-N	54% (5, 79)
Reduction in colposcopy exams	TVC-N	29% (22, 36)
Reduction in excisional procedures	TVC-N	70% (58, 79)

TVC-N means TVC naive: a TVC subset including women 15–25 years old who received ≥1 vaccine dose with normal cytology, seronegative for HPV-16/18 and DNA-negative for 14 oncogenic HPV types at baseline ((16, 18, 31. 33, 35, 39,45, 51, 52, 56, 58, 59, 66, and 68); case counting starting first day after first injection. Subjects were followed for an average of 39.5 months after first injection.

ASCUS means atypical squamous cells of undetermined significance irrespective of HPV type association.

LSIL means low-grade squamous intraepithelial lesion irrespective of HPV type association.

HSIL means high-grade squamous intraepithelial lesion irrespective of HPV type association.

**Table 6:** Vaccine efficacy in women 15–25 years old who were DNA-negative for HPV 16 or 18 at baseline [67].

Endpoint	Population	Serostatus	Vaccine efficacy (96.1% CI)	Average time of follow-up
6-month persistent infection caused by HPV 16/18	ATP-E	Seropositive	81% (59, 92)	41 month
12-month persistent infection caused by HPV 16/18	ATP-E	Seropositive	92% (64, 99)	41 month
CIN 2+ caused by HPV 16/18	ATP-E*	Seropositive or seronegative	99% (90, 100)	41 month

Infection definitions: 6-month definition required the detection of the same HPV type in two consecutive cervical samples, with no negative sample in between, over a minimum of 5 months; a 12-month definition required the detection of the same HPV type at consecutive assessments, with no negative samples in between, over a minimum of 10 months.

ATP-E means according to protocol for efficacy all women who met eligibility criteria and complied with the protocol, who received three injections; whose baseline Pap was normal, ASCUS, or LSIL cases counting starting day after the third vaccination.

ATP-E* means those subjects that are HPV-DNA-negative for HPV 16/18 at study entry regardless of initial serostatus with the HPV type assignment algorithm aand used to resolve causation when multiple HPV types were present. 14% of women were seropositive for HPV 16, and 10% were seropositive for HPV 18 at study entry; all were HPV-DNA-negative for HPV 16/18.

**Table 7:** Efficacy in less than 3-doses [68].

Endpoint	Population	Vaccine efficacy (95% CI)	Median time of follow-up
2 doses	TVC*	84% (50, 96)	4 years
1 dose	TVC*	100% (67, 100)	4 years

One-year persistence was defined as two positive tests for the same HPV type in visits 10+ months apart in women negative at enrollment for that HPV type with no intervening negatives and whose infection occurred after randomization.

TVC* means total vaccinated cohort of women who were HPV 16/18 DNA-negative at baseline regardless of serostatus and regardless of entry cytology and who had at least one follow-up datum.

Attack rate of HPV 16/18 infection in the control arm was consistently 4.5–5/100 women over the study.

**Table 8:** Factor of antibody titer increase over natural infection titers for each age group by oncogenic HPV type for Cervarix [69–72].

	10–15 years old [72]		15–25 years old [70, 71]		26–45 years old [71]		46–55 years old [71]	
	HPV 16	HPV 18	HPV 16	HPV 18	HPV 16	HPV 18	HPV 16	HPV 18
Month 7	253	170	107	82	230	91	84	57
Month 24	40	25	22	26	28	12	16	8
Month 48	29	18	20	15	18	8	11	5
Seroconversion at 48 months: % (95% CI)	100% (98, 100)	100% (98, 100)	100% (98, 100)	100% (98, 100)	100% (97, 100)	100% (98, 100)	100% (97, 100)	99.4% (97, 100)

Population is according to protocol, which included females seronegative to HPV 16/18 at baseline who received all three doses of study vaccine or placebo according to schedule, complied with the blood sampling schedule, and did not become positive for HPV-16/18-DNA during the trial.

Seropositivity at 48 months among women seronegative for HPV 16/18 at baseline, who received Cervarix in the ATP cohort. Seropositivity is defined as antibody titers ≥8 ELU/mL for HPV 16; ≥7 ELU/mL for HPV-18. Measured by type-specific ELISA testing.
